# Durability of Stavudine, Lamivudine and Nevirapine among Advanced HIV-1 Infected Patients with/without Prior Co-administration of Rifampicin: A 144-week Prospective Study

**DOI:** 10.1186/1471-2334-8-136

**Published:** 2008-10-14

**Authors:** Weerawat Manosuthi, Preecha Tantanathip, Wisit Prasithisirikul, Sirirat Likanonsakul, Somnuek Sungkanuparph

**Affiliations:** 1 Bamrasnaradura Infectious Diseases Institute, Ministry of Public Health, Nonthaburi, 11000, Thailand; 2 Faculty of Medicine Ramathibodi Hospital, Mahidol University, Bangkok, 10400, Thailand

## Abstract

**Background:**

To date, data on the durability of a regimen of stavudine, lamivudine and nevirapine are very limited, particularly from the resource-limited settings.

**Methods:**

A prospective cohort study was conducted among 140 antiretroviral-naïve patients who were enrolled to initiate d4T, 3TC and NVP between November 2004 and March 2005. The objectives were to determine immunological and virological responses after 144 weeks of antiretroviral therapy. Seventy patients with tuberculosis also received rifampicin during the early period of antiviral treatment (TB group).

**Results:**

Of all, median (IQR) baseline CD4 cell count was 31 (14–79) cells/mm^3^; median (IQR) baseline HIV-1 RNA was 433,500 (169,000–750,000) copies/mL. The average body weight was 55 kilograms. By intention-to-treat analysis at 144 weeks, the overall percentage of patients who achieved plasma HIV-1 RNA <50 copies/mL was 59.3% (83/140). In subgroup analysis, 61.4% (43/70) patients in TB group and 57.1% (40/70) patients in control group achieved plasma HIV-1 RNA <50 copies/mL (RR = 1.194, 95%CI = 0.608–2.346, *P *= 0.731). Eight (5.8%) patients discontinued d4T due to neuropathy and/or symptomatic lactic acidosis.

**Conclusion:**

The overall durability and efficacy of antiviral response of d4T, 3TC and NVP are satisfied and they are not different between HIV-1 infected patients with and without co-administration of rifampicin due to tuberculosis. However, stavudine-related adverse effects are concerns.

**Trial registration:**

ClinicalTrials.gov Identifier NCT00703898

## Background

Currently, the preferred first-line antiretroviral regimens use a combination of two nucleoside reverse transcriptase inhibitors (NRTIs) and either a non-nucleoside reverse transcriptase inhibitor (NNRTI) or a ritonavir-boosted protease inhibitor [1]. Although nevirapine is used as an alternative to efavirenz for initial regimen in developed countries, nevirapine has still been a key antiretroviral drug in many resource-limited countries including Thailand due to its accessibility and affordability. In addition, a component of stavudine and lamivudine is still widely used as a backbone in the antiretroviral regimen in this setting [2]. To date, data on the durability of a regimen of stavudine, lamivudine and nevirapine are very limited, particularly from the resource-limited settings.

In addition, HIV-1 infection has contributed to a significant increase in the worldwide incidence of tuberculosis and this has substantially affected the mortality [3,4]. Rifampicin is a key antituberculous drug. Unfortunately, rifampicin is associated with a significant drug interaction with nevirapine [5,6]. The results from previous studies have shown that nevirapine 400 mg/day-based regimen may be adequate to treat patients with tuberculosis and receiving rifampicin [7-11]. Herein, we continued the previously described prospective pharmacokinetic study [12] with the objectives to evaluate the treatment outcomes after 144 weeks of antiretroviral treatment (ART) regarding: (1) virological and immunological responses in all patients; and comparison of responses between the patients who received a regimen of stavudine, lamivudine and nevirapine alone (control group) and the patients with previous diagnosis of tuberculosis and concomitant receiving of rifampicin during the early period of ART (TB group) and (2) treatment outcomes of tuberculosis after 144 weeks of ART in TB group patient.

## Methods

The study design was a prospective cohort study involving 140 HIV-infected Thai patients in the Bamrasnaradura Infectious Diseases Institute, Ministry of Public Health, Nonthaburi, Thailand. There were equally 70 patients in TB group and control group. Initial enrollment was from November 2004 to March 2005 as previously described [12]. Inclusion criteria for the TB group were: (1) HIV-infected individuals ≥15 years of age, (2) diagnosed active TB by clinical features, positive acid-fast stain and/or positive culture for *Mycobacterium tuberculosis*, (3) receiving rifampicin-containing anti-TB regimen ≥1 month prior to enrollment, (4) CD4 cell count <350 cells/mm^3 ^and (5) willing to participate and give consent form. Inclusion criteria for the control group were: (1) HIV-infected individuals ≥15 years of age (2) not receiving RFP within 1 month prior to enrollment, (3) CD4 cell count <350 cells/mm^3 ^and (4) willing to participate and give consent form. Exclusion criteria for both two study groups was: (1) previous antiretroviral therapy, (2) pregnancy, (3) receiving a medication that has drug-drug interactions with NVP or RFP and (4) aspartate aminotransferase (AST) and alanine aminotransferase (ALT) >5 times of upper limit of normal range. The administered antiretroviral drugs were stavudine, lamivudine and nevirapine. All patients received NVP 200-mg once-daily lead-in dose for 14 days, prior to escalation to 200 mg twice daily. In the present study, the patients in both groups were followed up through 144 weeks in which period of time they were assessed clinically and evaluated for adverse events. CD4 cell counts and plasma HIV-1 RNA were assessed every 12 weeks until 96 weeks of ART and then every 24 weeks through 144 weeks. The patients in TB group were repeatedly tested for chest X-ray and clinically evaluated for tuberculosis at 144 weeks of ART. The institutional ethics committees of Bamrasnaradura Infectious Diseases Institute and Ministry of Public Health approved the study. All patients signed the inform consent.

The dosage of stavudine was adjusted by body weight (i.e., stavudine 30 mg and 40 mg twice a day for body weight ≤60 kg and >60 kg, respectively). The general characteristics (e.g., gender, age, body weight, body mass index (BMI), previous opportunistic infections and site of tuberculosis infection) were recorded. Blood samples were obtained to study CD4 cell counts by flow cytometry and HIV-1 RNA by polymerase chain reaction using Roche Amplicor^® ^version 1.5 (Roche Diagnostics, Branchburg, NJ, U.S.A.); lower limit of HIV-1 RNA detection, 50 copies/ml. The virological failure was defined as either a rebound plasma HIV-1 RNA of >1,000 copies/mL after having previously undetectable value or lack of achievement to <50 copies/mL at 24 weeks of ART. Genotypic resistance testing (TRUGENE HIV-1 Genotyping Assay, Visible Genetics Inc., Toronto, Canada) was performed after the patient was documented virological failure.

Anti-tuberculosis regimen included isoniazid, rifampicin, ethambutol and pyrazinamide were administered in the first two months followed by isoniazid and rifampicin for the subsequent four months. Tuberculosis treatment outcomes (cure, treatment completed, treatment failure) were evaluated by using definitions from WHO and the European Region of the International Union Against Tuberculosis and Lung Disease (IUATLD) [13].

Power and Sample Size version 1.01 [14] was used to calculate sample size by testing for equivalence of plasma NVP level as previously described [12]. A chi-square test was used to compare the number of patients who achieve undetectable HIV-1 RNA between groups. *P *value < 0.05 was considered as statistically significant. All analyses were performed using SPSS version 11.5.

## Results

**Figure 1:**
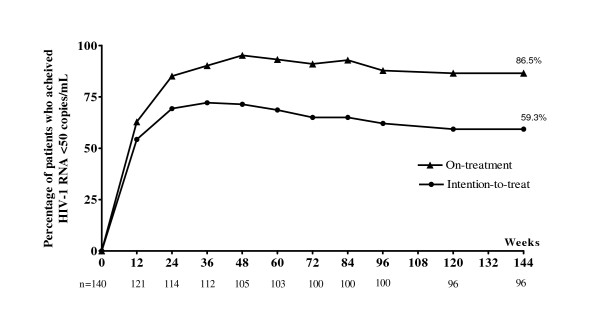
Proportion of patients who achieved undetectable plasma HIV-1 RNA <50 copies/mL.

**Figure 2:**
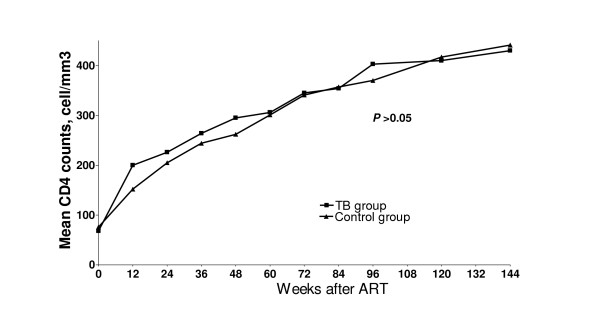
Mean CD_4 _cell count between TB and control groups.

A total of 140 patients were eligible and initially enrolled to the study. Seventy patients were equally included in each group. The patients' general characteristics, baseline CD4 counts and plasma HIV-1 RNA of each group are shown in table 1. Of 70 patients in TB group, 31 (44.3%), 20 (28.6%), 14 (20%), 3 (4.3%), 2 (2.9%) patients were diagnosed pulmonary tuberculosis, disseminated tuberculosis, cervical tuberculous lymphadenitis, gastrointestinal tuberculosis and tuberculous meningitis, respectively. By intention-to-treat analysis, the overall percentage of patients who achieved plasma HIV-1 RNA <50 copies/mL at 144 weeks was 59.3% (83 of 140). Figure 1 shows proportion of the patients who achieved undetectable plasma HIV-1 RNA at each time point by intention-to-treat analysis and on-treatment analysis. In subgroup analysis, 61.4% (43 of 70) patients in TB group and 57.1% (40 of 70) patients in control group achieved plasma HIV-1 RNA <50 copies/mL (RR = 1.194, 95%CI = 0.608–2.346, *P *= 0.731). Mean CD4 cell count response is shown in figure 2.

**Table 1:** Baseline characteristics of 140 HIV-infected patients

**Characteristics**	**Total *(n = 140)***	**TB Group *(n = 70)***	**Control Group *(n = 70)***	***P *value**
Gender: Male	95 (68%)	56 (80%)	39 (56%)	0.002

Age, years, mean ± SD	35.7 ± 7.6	34.4 ± 6.2	37.2 ± 8.7	0.028

Body weight, Kgs, mean ± SD	54.6 ± 9.6	54.7 ± 8.7	54.4 ± 10.6	0.827

Body mass index, mean ± SD	20.1 ± 2.9	19.7 ± 2.4	20.5 ± 3.4	0.097

CD4 cell counts, cells/mm^3^, mean ± SD	62 ± 74	61 ± 74	76 ± 75	0.823

%CD4, median (IQR)	5 ± 5	6 ± 5	5 ± 5	0.226

Plasma HIV RNA, copies/ml, median (IQR)	433,500 (169,000–750,000)	505,000 (269,000–750,000)	291,000 (94,600–714,000)	0.014

Plasma HIV RNA, Log copies/ml, median (IQR)	5.6 (5.2–5.9)	5.7 (5.4–5.9)	5.6 (5.0–5.9)	0.004

ALP, mg/dl, median (IQR)	98 (71–142)	110 (75–154)	91 (70–128)	0.072

AST, U/l, median (IQR)	30 (20–50)	36 (27–60)	35 (26–47)	0.371

ALT, U/l, median (IQR)	27 (19–42)	27 (18–51)	32 (22–49)	0.169

Total bilirubin, mg/dl, median (IQR)	0.6 (0.4–0.7)	0.6 (0.5–0.8)	0.5 (0.4–0.7)	0.051

IQR = interquartile range

**Figure 3:**
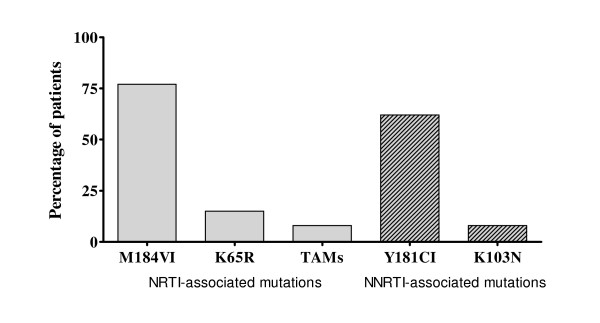
Drug resistance mutations in patients with virological failure.

Of all, 57 patients needed to discontinue ART after 144 weeks of ART, 27 patients were in TB group and 30 patients were in control group. There was no difference in term of ART discontinuation between the two groups (*P *= 0.688). The reasons were as follow: lost to follow-up (14 patients, 10.0%), HIV drug resistance (13, 9.3%), NVP-related skin rashes grade II-III (11, 7.9%), d4T-related neuropathy and/or symptomatic lactic acidosis (8, 5.8%), deceased (7, 5.0%), transferred care (3, 2.1%) and drug interaction due to receiving itraconazole (1, 0.7%), respectively. Of 70 patients in each group, 10% (7 of 70) and 9% (6 of 70) patients in the TB group and control group developed HIV-1 RNA >1,000 copies/mL, respectively (*P *= 1.000). Among 13 patients with drug resistance, 5 (38%) had mutations contributed to only NRTI resistance; 1 (8%) had mutations contributed to only NNRTI resistance; and 7 (54%) patients had mutations contributed to both NRTI and NNRTI resistance. For NRTI-resistance mutations, M184V/I was the most common (10 of 13, 77%). Thymidine analogue associated mutations (TAMs) were found in 1 (8%) patients. K65R was observed in 2 of 13 (15%) patients. For NNRTI-resistance mutations, there were Y181C/I (8 of 13, 61%) and K103N (1 of 13, 8%) as shown in figure 3. There were no differences in terms of NRTI and NNRTI-resistance associated mutations between the two subgroups (*P *> 0.05).

In term of treatment outcomes of 70 patients in TB group, 55 (79%) patients were cured or completed treatment; 7 (10%) lost to follow-up; 5 (7%) deceased (from MAC infection, TB paradoxical reaction and severe wasting for each patient); 2 (3%) transferred care; and 1 (1%) had relapse.

## Discussion

The present study has demonstrated that an antiretroviral regimen of stavudine, lamivudine and nevirapine showed acceptable long-term antiviral effectiveness in advanced HIV-1 infected patients. By intention-to-treat analysis, approximately 60% patients still achieve undetectable plasma HIV-1 RNA through 144 weeks. Consequently, CD4 cell count continues to increase significantly over the study period. This is considered favorable outcome when compared to the other NNRTI-based regimen [15-17]. In subgroup analysis, we can not find any discrepancy of antiviral response between HIV-infected patients who previously received rifampicin and those who did not received rifampicin. This confirms the previous published short-term efficacy data in these patients [12,18]. Average mean trough plasma nevirapine levels of week 8 and week 12 was 5.40 mg/l in the TB group and 6.56 mg/l in control group (*P *= 0.048) [12]. Although minimum plasma concentration of nevirapine is somewhat different during the early period of ART between the two subgroup patients as mentioned, the long-term antiviral responses between these two groups were not different as demonstrated in the present study. In addition, the prevalence of Y181C/I, K103N and other mutations that confer resistant to NNRTIs were not different between the two groups when virological failure was detected. However, therapeuric nevirapine level may be needed to verify in the further study.

Long-term acceptability and safety may be a concerned issue for this regimen. It is well established that long-term administration of NRTI, particularly stavudine, can cause mitochondrial toxicity. The clinical manifestations of this adverse effect present as hyperlactatemia and polyneuropathy [19]. In the present cohort, 5.8% of patients subsequently developed stavudine-associated toxicity and needed to substitute stavudine with other NRTI. A 2008 ART guideline that developed by the Department of Health and Human Services (DHHS) panel no longer recommends the component of stavudine combined with lamivudine as a backbone NRTIs for initial therapy in treatment-naïve patients [1]. Thus, zidovudine and tenofovir could be the options in this area. Nevertheless, the frequency and severity of adverse events associated with the use of zidovudine are greater in patients with advanced disease at the time of initiation of therapy [20]. Likewise, tenofovir is more expensive and less available. This brings to a concern of an appropriate backbone NRTI in the first-line regimen in resource-limited countries. To date, World Health Organization (WHO) has not yet updated its guidelines for the use of ART in the countries with constrained resources since 2006 [21]. According to these limitations and until the other options are more accessible; stavudine is still a component drug in the treatment guidelines for the resource-limited countries. Conversely, strategic treatment to minimize long-term toxicity of stavudine, such as switching to other drugs at an optimal timing, should be evaluated further.

The initial sample size of the present study is calculated from the difference of minimum plasma concentration of nevirapine between the two groups of patients [12]. Thus, it may not be enough to detect the difference of long-term antiviral responses. Another limitation is that many studies showed the impact of genetic polymorphism on NVP metabolism [22,23]. Thus, this result should be cautiously applied with the other ethnic population.

## Conclusion

In conclusion, a regimen of stavudine, lamivudine and nevirapine provides the satisfied durability and immunological response in very advanced HIV-infected patients. There is no difference of the 144-week efficacy between HIV-1 and tuberculosis co-infected patients receiving rifampicin and HIV-1 mono-infection not receiving rifampicin. However, long-term safety of stavudine is a concern. Relapse rate after 3 years of initiation of tuberculosis treatment in the patients who are receiving ART is low. In resource-limited settings, a regimen of stavudine, lamivudine and nevirapine is still an important first-line option for advanced HIV-1 infected patients. Strategy to minimize long-term toxicity of stavudine, such as switching to other drugs at an optimal timing, should be evaluated further.

## Competing interests

The authors declare that they have no competing interests.

## Authors' contributions

WM participated in the design of the study, draft the manuscript and performed the statistical analysis. PT participated in its design and coordination. SL carried out the laboratory testing. SS participated in draft the manuscript and statistical analysis.

## Pre-publication history

The pre-publication history for this paper can be accessed here:


